# Complex shaped periodic corrugations for broadband Bull’s Eye antennas

**DOI:** 10.1038/s41598-021-94232-2

**Published:** 2021-07-20

**Authors:** Despoina Kampouridou, Alexandros Feresidis

**Affiliations:** Department of Electronic, Electrical and Systems Engineering, University of Birmingham, Birmingham, B15 2TT UK

**Keywords:** Electrical and electronic engineering, Applied physics

## Abstract

Periodically corrugated metallic antennas have been developed in recent years from microwave to THz frequencies, due to their advantages of highly directive radiation patterns, low profile and ease of fabrication. However, the limited gain bandwidth of such antennas remains one of their inherent disadvantages. In terms of design, the majority of the existing implementations in literature utilize the standard rectangular shaped corrugated unit cell. In this paper, we propose novel complex shaped corrugated unit cells that produce a broadband performance when assembled in a periodic configuration. Two broadband prototypes are presented at the Ku frequency band that are formed of hybrid shaped corrugations. The first prototype of six periodic rings achieves, for the first time, a flat gain simulated response with a maximum value of 15.7 dBi, 1-dB gain bandwidth of 16.4%, and an extended 3-dB gain bandwidth of 19.64%. The second novel prototype of five rings achieves an enhanced 3-dB gain bandwidth of 15.2% and maximum gain of 18.1 dBi.

## Introduction

In recent years, there has been a significant research interest in a family of planar corrugated metal antennas (Bull’s Eye antennas) that radiate a via subwavelength aperture etched off the centre of the metallic structure, with high gain radiation patterns^1–3^. These antennas are directly scalable from microwave to low THz frequencies and offer the advantages of high directivity, very low profile (less than a wavelength) and easy design and fabrication process^4^.

Since the first Bull’s Eye antenna prototype was introduced by Beruete et al.^4^, several designs have emerged in literature with optimized performance in various aspects. An improvement in efficiency was achieved by introducing a sinusoidally modulated corrugated surface^5^, while in other implementations the matching bandwidth was significantly extended with the use of an open and, next, a tapered waveguide aperture instead of the subwavelength slot^6^. Other configurations include the superposition of a dielectric layer on top of a corrugated surface^7^, or a double layer corrugated antenna^8^, both achieving an increased directivity. Most of these implementations, however, suffer from a narrowband gain performance.

In this work, the typically limited gain bandwidth of corrugated metallic antennas is expanded by introducing novel concepts of corrugated unit cells. So far, the most referenced corrugated unit cell has been the rectangular shaped, although some more have been investigated, such as triangular shaped^9^ or sinusoidal^5^, but without remarkable effects on the antenna bandwidth. Our proposed unit cell designs create more than one resonant gaps within a periodicity and avoid a topology that would increase the overall antenna size or fabrication complexity (extra layers or sinusoidal corrugations). A broadband, high gain antenna performance is thus achieved by the complex shaped unit cells only.

**Figure 1:**
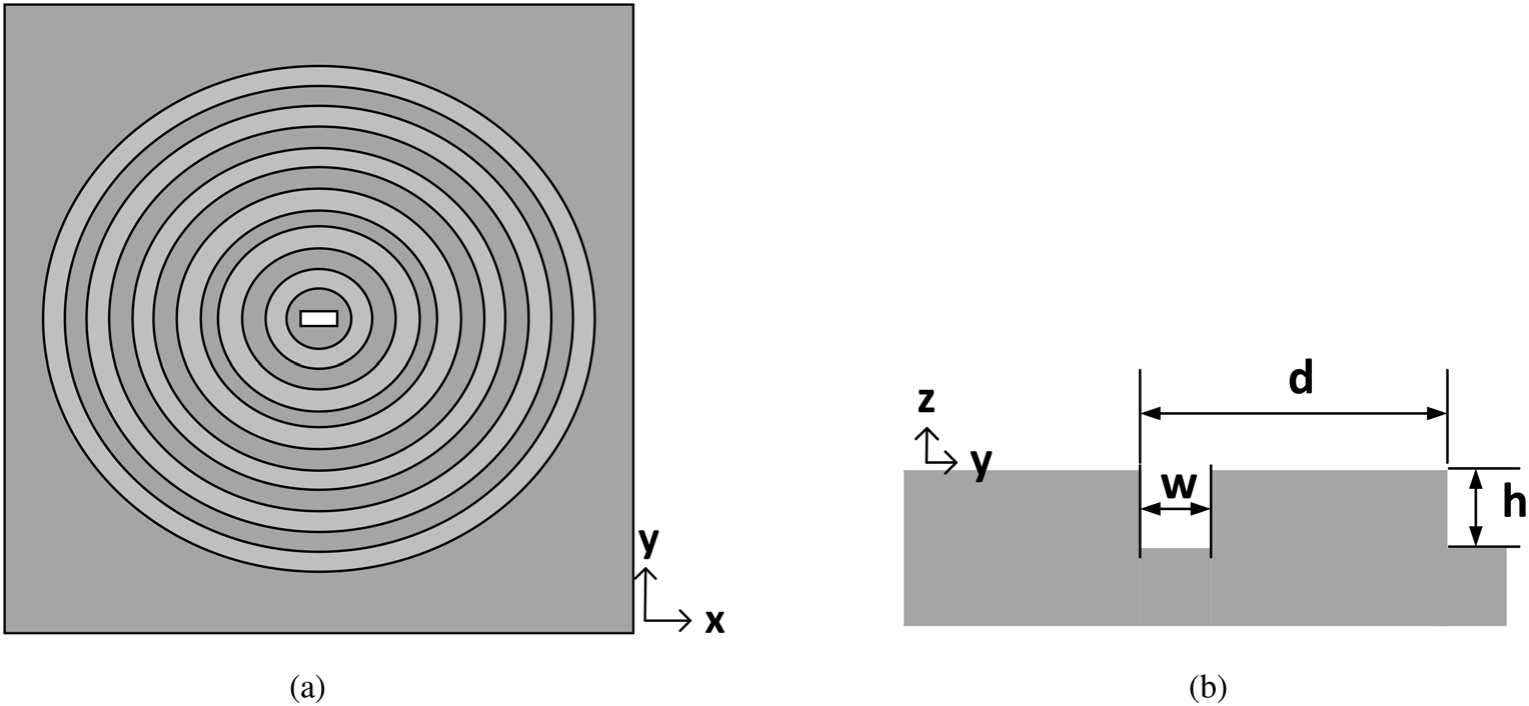
Standard Bull’s Eye antenna with $N=6$ rings. (**a**) Front view. (**b**) Cross section of a unit cell with design details.

**Figure 2:**
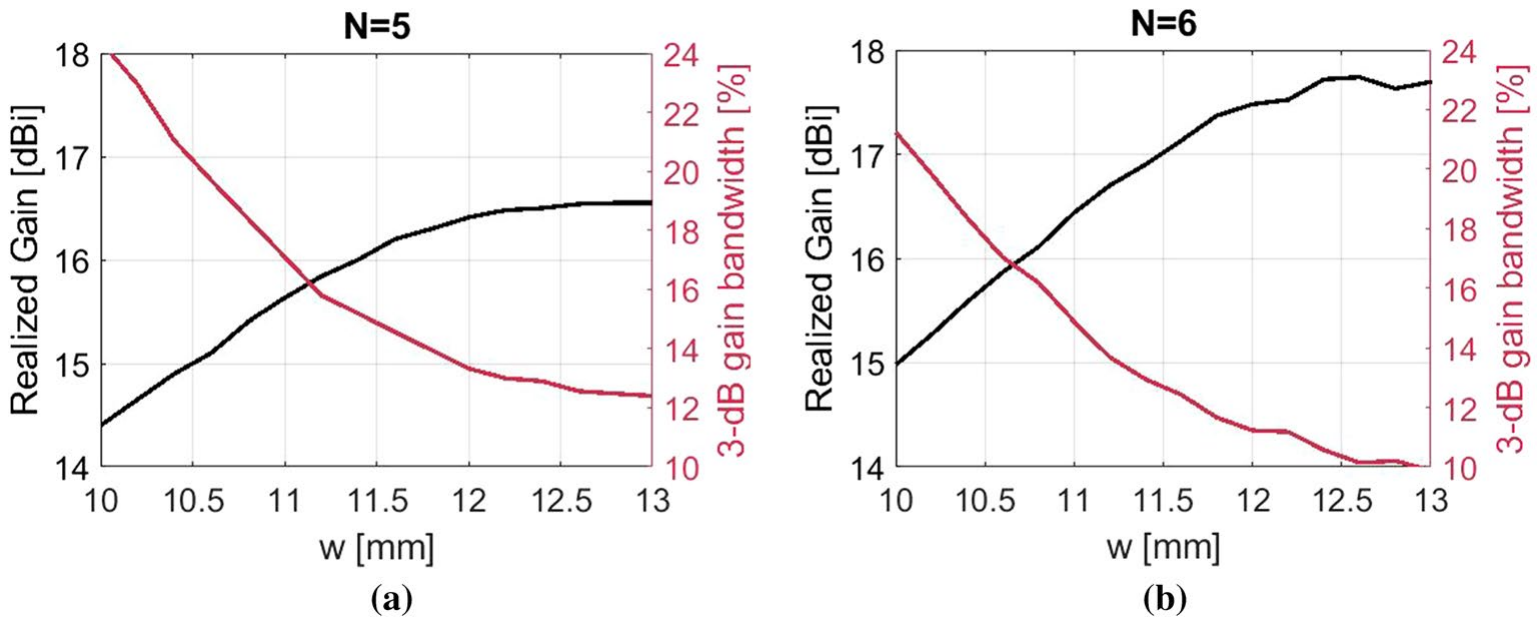
Simulation study over the relationship between gap width *w*, maximum realized gain and fractional gain bandwidth for a standard Bull’s Eye antenna of (**a**) $N=5$ and (**b**) $N=6$ rings.

In particular, two different corrugation concepts are introduced with distinct effects on the farfield performance of the finite size antenna; the perturbed corrugation, which consists of a metallic obstacle inside the primary unit cell gap, and the double corrugation, which employs a “corrugation within corrugation” concept. The effect of each of these new corrugation shapes on the antenna realized gain and bandwidth is examined separately. Next, the two aforementioned types of unit cells are combined into a hybrid unit cell that exploits both effects. Two optimized prototypes are extracted from this design process of hybrid corrugated cells. The first proposed model of six rings achieves a maximum gain of 15.7 dBi and a 1-dB gain bandwidth of 16.4%. The 3-dB gain bandwidth of this antenna is extended to 19.64%, compared to an antenna of the same size and similar gain. The second prototype of five rings achieves a maximum gain of 18.1 dBi, along with an extended 3-dB gain bandwidth of 15.2%. Both antennas outperform standard Bull’s Eye antennas of similar size and gain.

## Results

### The Impact of Rectangular Gaps

In this section, two Bull’s Eye antennas of different number of rings are examined as in Fig. 1, in order to demonstrate the relationship between the gap width *w*, the realized gain and the 3-dB gain bandwidth. The antenna design parameters have been optimized according to the guidelines for enhanced transmission of power as^10^:

1 $$\begin{aligned}&d \approx \lambda _{0} \\&h< \frac{(2k+1) \lambda _{0}}{4} \\ \end{aligned}$$

with $\lambda _{0}$ the free-space wavelength, *d* the periodicity and *k* a non-negative integer. For operation at the Ku band ($\lambda _{0}$ around 15 GHz), periodicity *d* is set to 19.6 mm and the depth of rings *h* is set to 3.31 mm. The antenna is fed with an open-ended waveguide aperture (equal to the WR-62 dimensions), in order to secure a good matching performance across the operational frequency band^6^. For a fixed periodicity *d*, a parametric sweep with respect to the gap width *w* is performed in CST Microwave Studio, tracking the maximum realized gain and 3-dB gain bandwidth of the antenna. Figure 2 shows the results of this parametric study.

**Figure 3:**
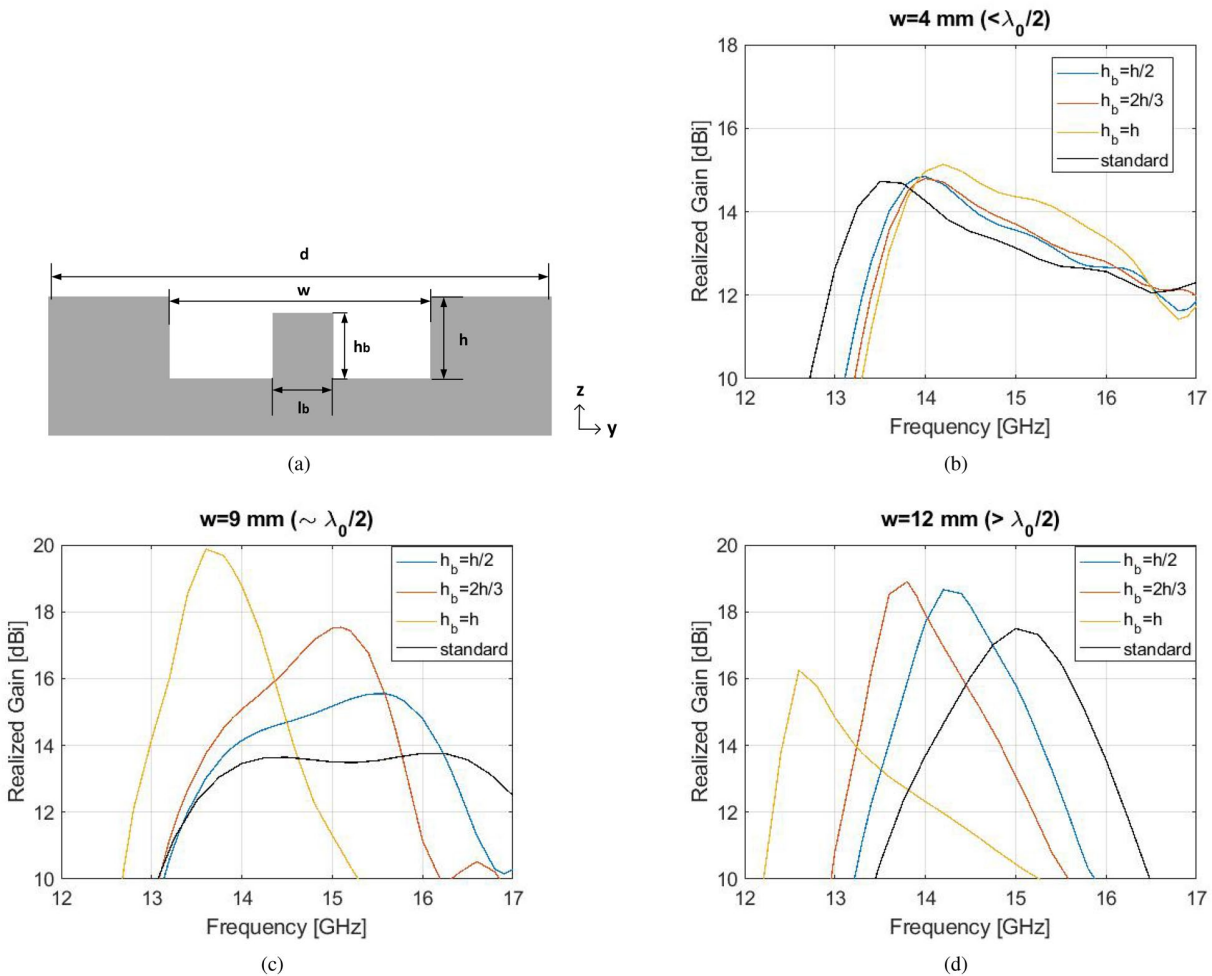
(**a**) Cross section of the perturbed corrugated unit cell. Simulated realized gain of the perturbed corrugated antenna of $N=6$ when (**b**) $w<\lambda _{0}/2$, (**c**) $w \sim \lambda _{0}/2$, (**d**) $w>\lambda _{0}/2$.

Only values of *w* over $\lambda _{0}/2$ are simulated, since it is expected that over this limit, the gap will excite the $TM_{1}$ mode of the assumed parallel-plate waveguide, and therefore achieve a low attenuation constant that can lead to a high gain performance of the antenna^11,12^. Antennas of gaps smaller than $\lambda _{0}/2$ operate with the fundamental *TEM* mode of the assumed parallel-plate waveguide, which has been reported of a high attenuation constant for such corrugated structures^13^. With respect to the depth of corrugations *h*, it has been observed that with deeper corrugations, a higher peak gain can be obtained, that results nonetheless in a more narrowband performance^3,14^. Therefore, the selected *h* of this implementation is a good compromise between high gain and satisfactory 3-dB gain bandwidth and is not subject to optimization.

Figure 2 presents the simulated results of two corrugated antennas of different numbers of rings ($N=5,6$), for gap widths between 10 mm (0.5$\,\lambda _{0}$) and 13 mm (0.65$\,\lambda _{0}$). Up to 11.7 mm (0.58$\,\lambda _{0}$) there is a continuous drop of the 3-dB gain bandwidth by 10%, while the realized gain is increased by 2 dBi. For $w> 11.7$ mm, the maximum gain reaches a saturation point of 16.6 dBi (with 12.3% corresponding bandwidth) for the $N=5$ antenna and 17.7 dBi (9.4% bandwidth) for the $N=6$ antenna. For $w>0.65\,\lambda _{0}$, the achieved maximum gain slightly drops as the 3-dB bandwidth continues to shrink. Therefore, as a compromise between high gain and satisfactory gain bandwidth, the antenna should be designed with gap widths up to 0.65$\,\lambda _{0}$. The numerical results of this section will be used as a reference standard Bull’s Eye antenna in the following sections, where novel types of corrugations are studied.

**Figure 4:**
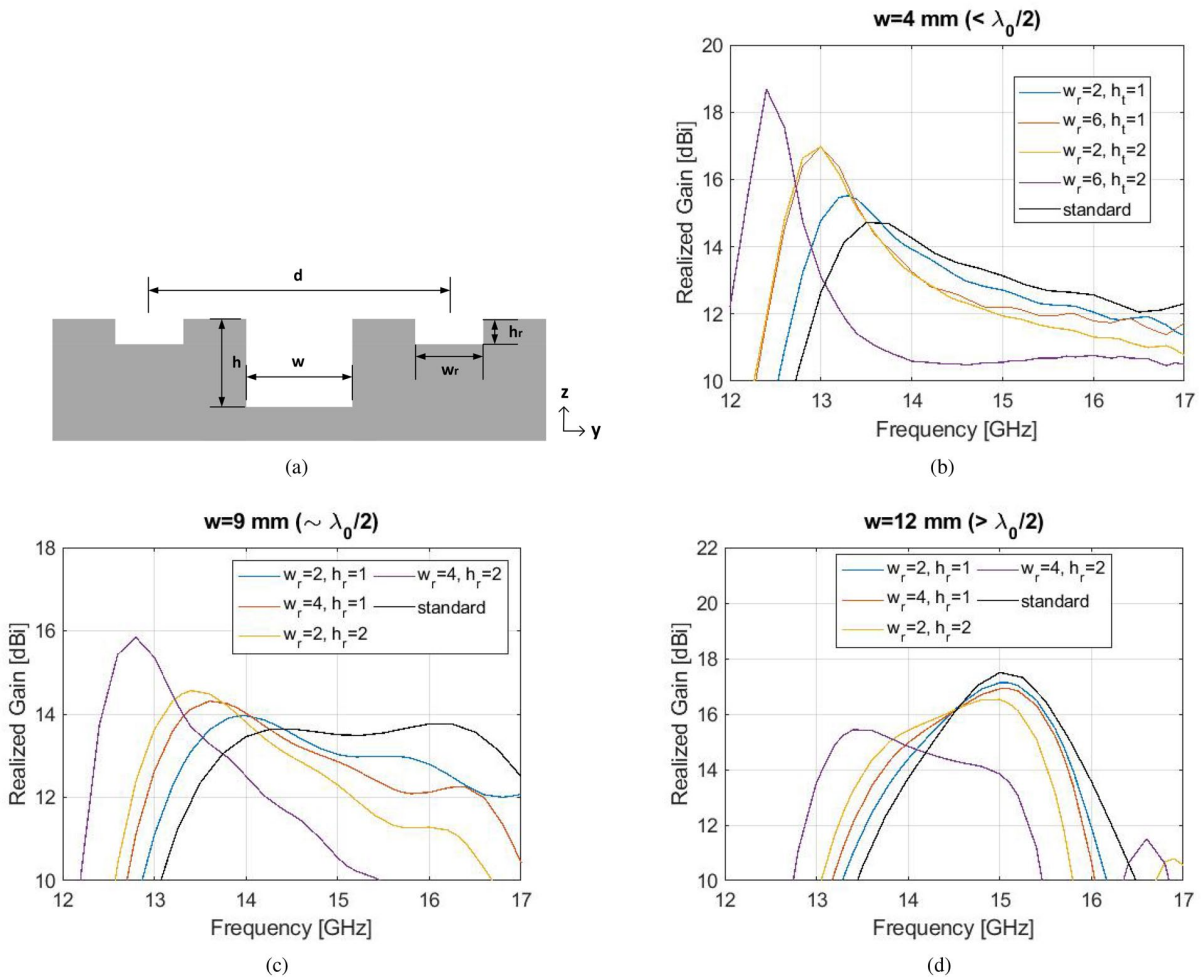
(**a**) Cross section of the double corrugated unit cell. Simulated realized gain of the double corrugated antenna with $N=6$ when (**b**) $w<\lambda _{0}/2$, (**c**) $w \sim \lambda _{0}/2$, (**d**) $w>\lambda _{0}/2$. Dimensions are in mm.

### Complex shaped corrugation concepts

The simulation studies of Fig. 2 demonstrate an upper limit in the performance of the standard corrugated designs of a fixed size, without many degrees of freedom for the optimization of their performance. It has also been demonstrated that an increase in the depth of corrugations *h* increases the gain, but consequently limits the gain bandwidth^3,14^. In this section, novel shapes of rectangular corrugations are proposed which consist of more than one resonant gaps within a periodic unit cell and contribute to a broadband gain performance.

#### The perturbed corrugation

The proposed perturbed unit cell configuration is introduced in Fig. 3a. In the middle of the central gap (*w*), a metallic perturbation of height $h_{b}$ and length (along *y*) $l_{b}$ is inserted. The concept of this engineering process is to increase the overall longitudinal resonant path by creating a second parallel plate waveguide inside the main gap (*w*). For direct comparison with the unit cell of Fig. 1b, the dimensions the unit cell of Fig. 3a are selected as $d=19.6$ mm, $h=3.3$ mm for operation at around 15 GHz. The rest of the parameters *w*, $h_{b}$ and $l_{b}$ are subject to change and their the effect is examined separately. A Bull’s Eye antenna with $N=6$ perturbed rings is studied in parametric sweep, with respect to the height of perturbation $h_{b}$. The size of this antenna is $24 \times 24 \times 0.75$ cm$^3$. The length $l_{b}$ is set arbitrarily to 1 mm ($\ll \lambda _{0}$). Three perturbed shaped corrugated antennas are simulated, with $w<\lambda _{0}/2$, $w \sim \lambda _{0}/2$ and $w>\lambda _{0}/2$ respectively. Their simulated realized gain performance is depicted in Fig. 3b–d for each *w* case.

**Figure 5:**
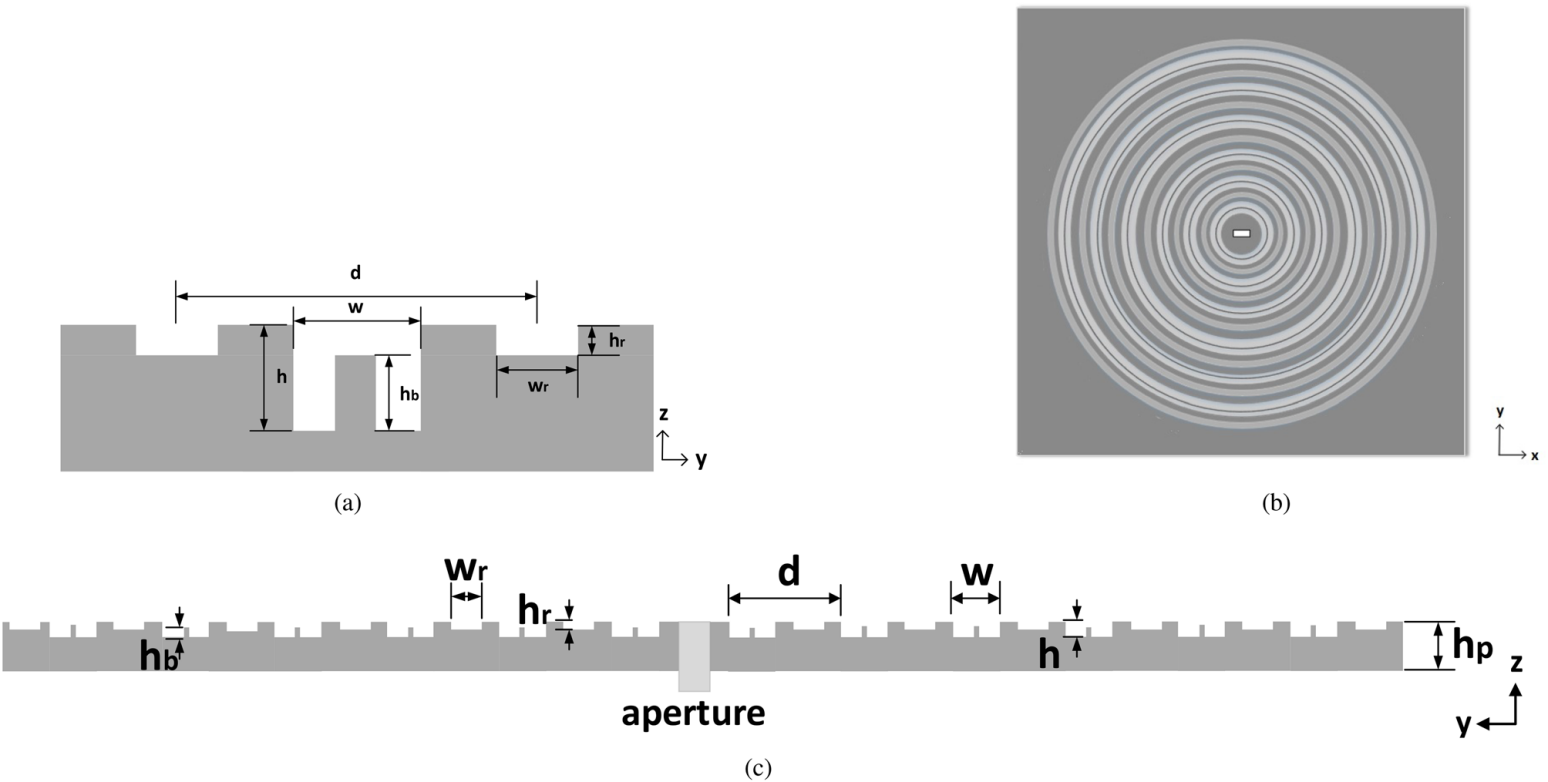
(**a**) Cross section of the hybrid corrugated unit cell. (**b**) Front view of the hybrid shaped antenna ($N=6$). (**c**) Profile view of the hybrid shaped antenna.

The realized gain of the antenna with $w<\lambda _{0}/2$ is not significantly affected by the presence of the perturbed corrugations, except from the small shift towards higher frequencies. When $h_{b}=h$ (with maximum realized gain 15 dBi, similar to the standard antenna with $h_{b}=0$), an extended 1-dB gain bandwidth is observed between 13.8 GHz and 15.3 GHz. In Fig. 3c,d, a significant enhancement in realized gain is achieved, along with slightly enhanced 3-dB gain bandwidth for some $h_{b}$ values. In particular, in Fig. 3c, the realized gain reaches up to 20 dBi for $h_{b}=h$, compared to only 13.8 dBi achieved by the standard antenna. When $h_{b}=h/2$, the maximum realized gain is 15.5 dBi with a 3-dB bandwidth of nearly 19.5%, while from a standard Bull’s Eye antenna of $N=6$, the same maximum gain is met with 18.9% corresponding bandwidth (Fig. 2b). Furthermore, when $h_{b}=2h/3$ and maximum realized gain is 17.5 dBi with a 3-dB gain bandwidth of 12.6%, a standard Bull’s Eye antenna of the same size achieves this realized gain with 11.3% bandwidth (Fig. 2b). The complex shaped antenna outperforms the standard antenna of $N=5$ (Fig. 2a) as well.

In Fig. 3d, a peak realized gain when $h_{b}=2h/3$ a peak gain of 19 dBi is achieved. In this case, however, the increase is no more than 1.5 dBi compared to the standard design. This is due to the dominant $TM_{1}$ mode, which is already fully formed inside a gap with $w>\lambda _{0}/2$, and is characterized by low attenuation constant^12^.Additionally, we observed that when $h_{b}=h$, the $S_{11}$ is deteriorated, which causes a difference of several dBis between realized gain and directivity. Directivity for this case is 21.5 dBi. Regarding the parameter $l_{b}$, simulations showed that it does not affect the farfield performance, therefore it can be fixed to an arbitrary value, as long as it is much smaller than $\lambda _{0}$. In conclusion, the first elements towards the bandwidth enhancement of Bull’s Eye designs are offered with these proposed perturbed corrugations when $h_{b}$ is selected between *h*/2 and 2*h*/3, and when the main gap *w* is at the order of $\lambda _{0}/2$.

**Figure 6:**
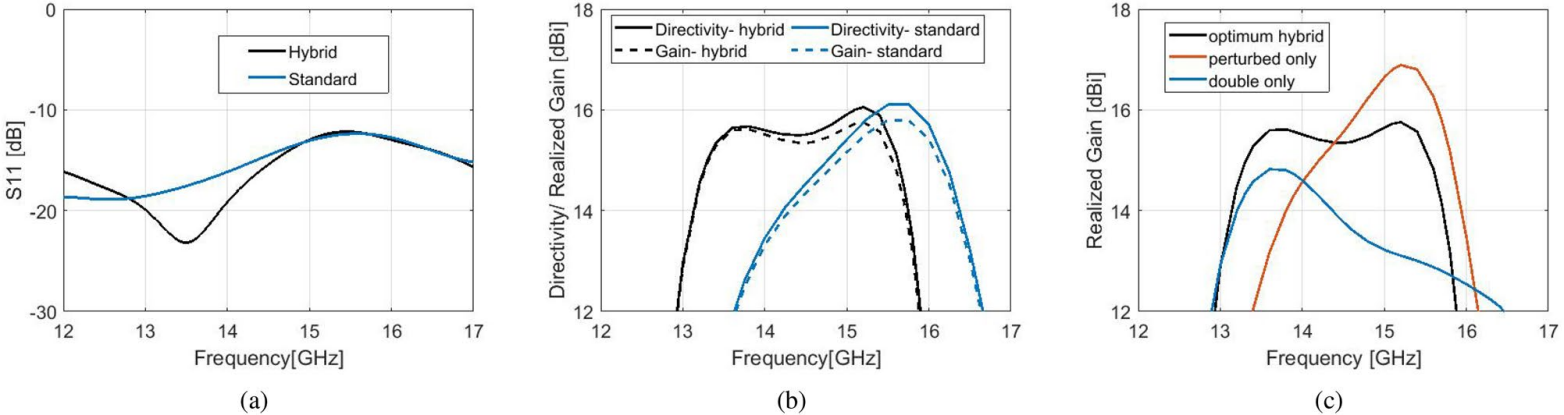
Simulated results of the optimum hybrid Bull’s Eye antenna with flat gain compared to a standard Bull’s Eye antenna ($N=6$) of similar gain. (**a**) $S_{11}$. (**b**) Directivity and realized gain. (**c**) Contribution of each corrugation type in the hybrid effect.

**Figure 7:**
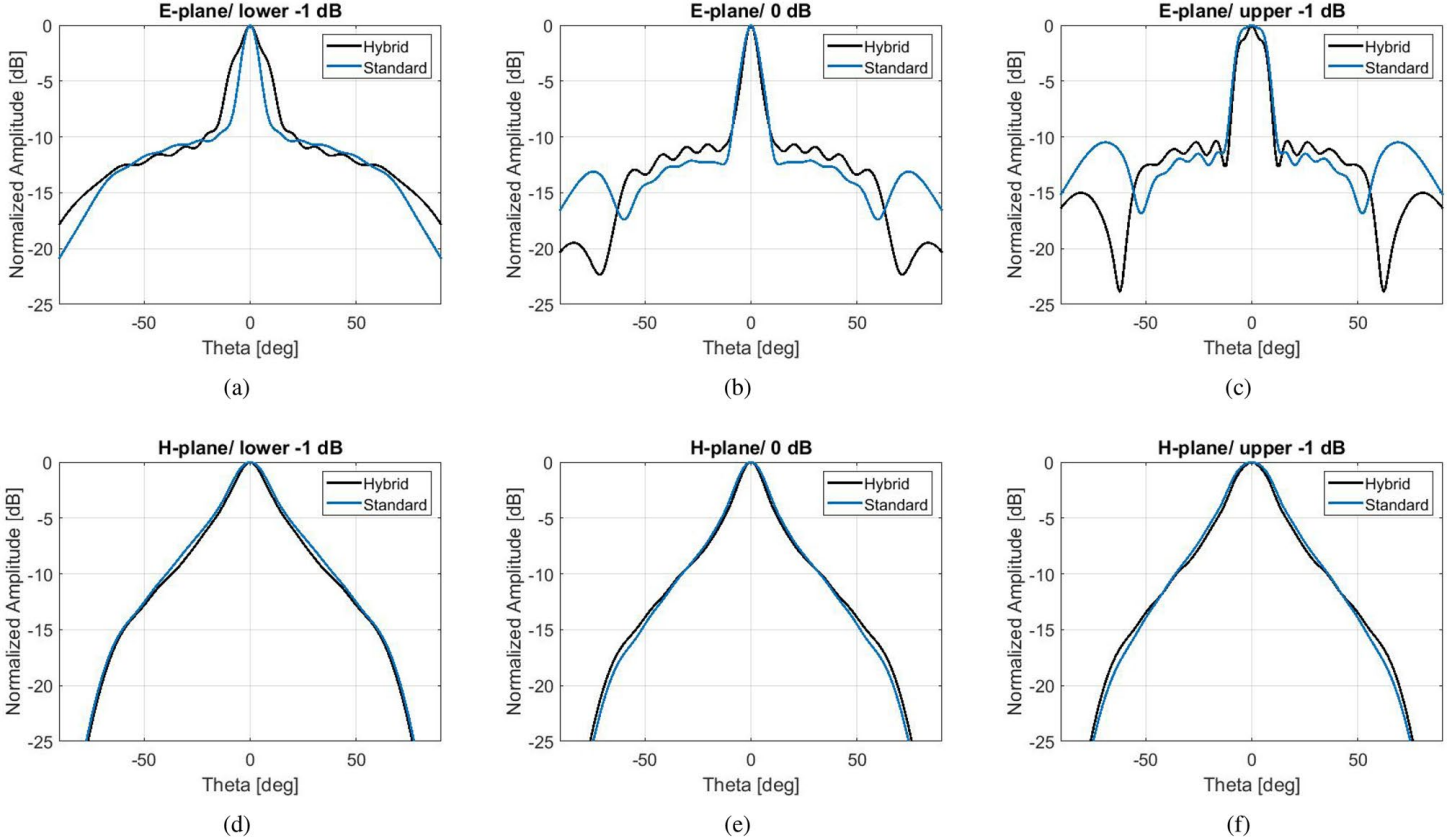
Simulated radiation patterns of the hybrid antenna with flat gain and the standard antenna of the same maximum gain.

#### The double corrugation

A novel “corrugation within corrugation” concept is proposed, or “double corrugation”, towards the goal of bandwidth enhancement, with parametric design details in Fig. 4a. A secondary corrugation is formed within a standard shaped unit cell, with length along *y* as $(d-w)/2$, gap $w_{r}$ and depth $h_{r}$. The simulated effect of the secondary gap with $w_{r}, h_{r}$ on a finite size antenna of $N=6$ is presented in Fig. 4b–d. All other antenna dimensions are the same as with Fig. 1. The size of this antenna is $24 \times 24 \times 0.75$ cm$^3$ and the feeding aperture is an open-ended waveguide (WR-62).

The effect of the secondary corrugation ($w_{r}$, $h_{r}$) is significant for $w \le \lambda _{0}/2$. The realized gain increases with $h_{r}$, with its peak shifted towards lower frequencies. In particular, when $w<\lambda _{0}/2$, the enhancement in realized gain is up to 4 dBi compared to the standard Bull’s Eye antenna, for the parameter set of ($w_{r}=6$ mm, $h_{r}=2$ mm). When $w \sim \lambda _{0}/2$, a maximum realized gain of 15.9 dBi is achieved, for $w_{r}=4$ mm and $h_{r}=2$ mm. The effect of the double corrugation is not significant when $w>\lambda _{0}/2$, which can be easily explained by the strong $TM_{1}$ mode dominating inside an electrically large gap *w*^12^. The double corrugation effect is stronger at lower frequencies when $h_{r}$ is larger. This shift can be explained from Eq. 1, where a maximum radiation is achieved at lower frequencies as the depth of corrugations increases.

#### The hybrid corrugation

The novel perturbed and double corrugated unit cell concepts are now combined into a hybrid form of corrugated unit cell, just as in Fig. 5a. This hybrid concept takes advantage of both distinct effects in directivity (gain) and gain bandwidth. It has been proven already that the perturbed effect is remarkable only when $w \ge \lambda _{0}/2$ and the double corrugation when $w \le \lambda _{0}/2$. Therefore, the design process of a hybrid corrugated unit cell is initiated with *w* around $\lambda _{0}/2$.

#### Optimum hybrid antenna with flat gain response (optimum A)

The first proposed optimum prototype of this work is a hybrid Bull’s Eye antenna of $N=6$ radiating via an open-ended waveguide aperture. For the first time, a flat gain (directivity) plot over frequency is achieved when the gain is high (over 14 dBi), along with an extended fractional 3-dB gain bandwidth. The optimized parameters of the proposed antenna are $w_{r}=8$ mm, $h_{r}=1$ mm, $h_{b}=1.64$ mm, $w=9.4$ mm, $h=3.31$ mm, $l_{b}=1$ mm.

A proper comparison is shown between the proposed model and a standard Bull’s Eye antenna of the same size and similar gain, which according to Fig. 2b is designed with $w=10.6$ mm (and *d*, *h* same with the hybrid model). Simulated results can be viewed in Fig. 6 for $S_{11}$, directivity and realized gain. It is evident that the satisfactory $S_{11}$ response of the open-ended waveguide is maintained in the hybrid design (Fig. 6a). The maximum directivity of both designs is 16 dBi (realized gain 15.7 dBi).The extraordinary characteristic of the novel hybrid design is the large 1-dB gain bandwidth, which covers the frequency range from 13.2 to 15.6 GHz (16.4%). Such a flat gain response cannot be achieved for corrugated antennas with high gain or directivity (over 14 dBi). For example, a flat gain response for a standard Bull’s Eye antenna is observed in Fig. 3.b, however its maximum value is only 13.8 dBi. From Fig. 6b, the 1-dB gain bandwidth of the standard design is only 9.1%. The 3-dB gain bandwidth of the hybrid model is at 19.64%, while for the standard antenna of $N=6$ it is 17.47%. The 3-dB gain bandwidth of the 5-ring antenna of similar gain is even less, around 15.5%.

The effect on each corrugation in the performance of the hybrid antenna can be viewed separately in Fig. 6c. The optimum model is compared with two antennas of the same size; the first antenna consists of perturbed corrugations only and the second antenna consists of double corrugations only, with the same dimensions as with the optimum hybrid design. The flat gain response of the hybrid model exhibits two peaks, each generated by the different component of the hybrid corrugated unit cell. The peak of maximum gain (15.7 dBi) is generated by the perturbed corrugations, at 15.2 GHz, and the second peak that maintains the flat gain (15.6 dBi) at 13.6 GHz is generated by the double corrugations.

**Figure 8:**
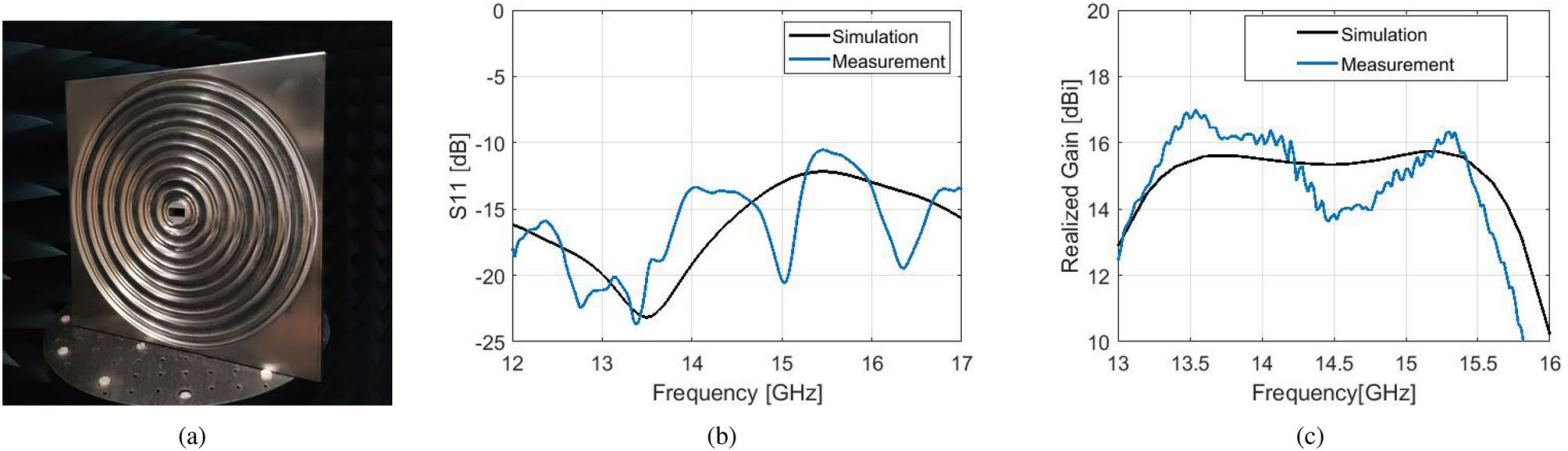
(**a**) Fabricated prototype of the hybrid corrugated antenna with flat gain response. (**b**) Measured $S_{11}$ versus simulated. (**c**) Measured gain versus simulated.

**Figure 9:**
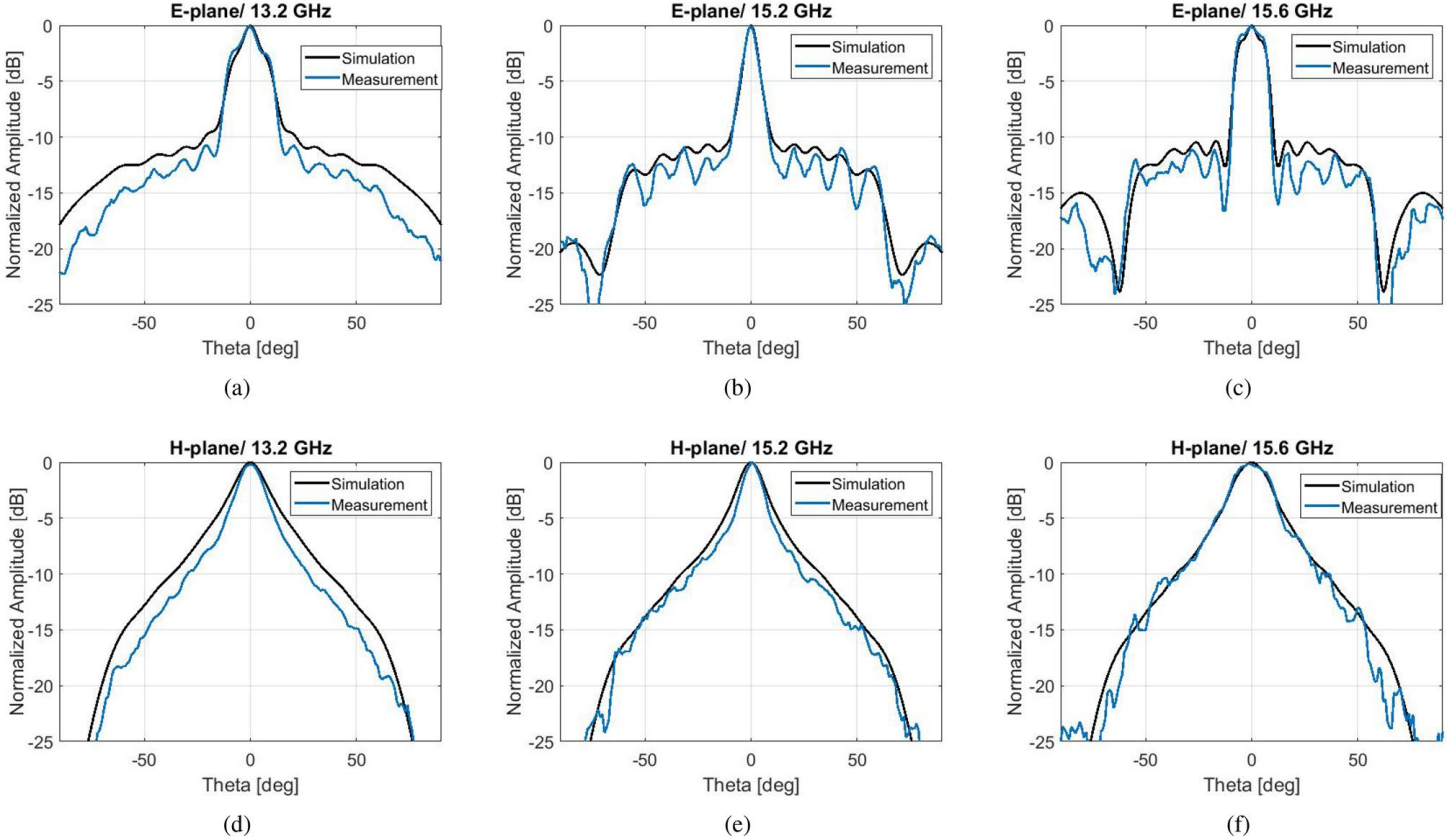
Measured radiation patterns of the hybrid antenna with flat gain and comparison with simulated.

The simulated radiation patterns are shown in Fig. 7, at the frequencies which determine the 1-dB gain spectrum. The *H*-planes are not affected by the hybrid effect. At the frequency of maximum gain, the side lobe level of the *E*-plane is at − 10 dBi for the hybrid antenna and − 12 dBi for the standard antenna, maintaining a highly directive lobe. At bigger angles (over $\pm 60^{\circ }$) the side lobe level of the hybrid antenna is significantly suppressed by more than 5 dBi compared to the standard antenna at the frequencies of maximum directivity and upper − 1 dB, and about 4 dBi at the frequency of lower -1 dB. At the lower limit of the 1-dB spectrum, however, the standard design exhibits a narrower main lobe.

For an experimental validation, a prototype of this hybrid corrugated antenna with flat gain response has been fabricated with CNC milling technique at the University of Birmingham, seen in Fig. 8. The measured results for the $S_{11}$ and realized gain can be viewed in Fig. 8b,c. A broadband $S_{11}$ performance below − 10 dB is achieved between 12 and 17 GHz, in good agreement with simulations. The peak realized gain of approximately 16.7 dBi is obtained at 13.4 GHz. It is evident that there have been fabrication errors in the process which result in a lower gain response of about 1.5 dBi between 14.3 and 15 GHz. Still, the overall measured gain performance remains between the expected 3-dB gain bandwidth. Figure 9 depicts the normalized measured patterns on both *E* and *H* planes, at the frequencies which define the 1-dB gain spectrum of the simulated model. The agreement between simulations and measurements is overall very satisfactory. At 13.2 GHz the measured pattern exhibits lower side lobes than the simulated. This is due to the higher measured gain of the fabricated antenna at the frequency band between 13.2 and 13.8 GHz, due to fabrication intolerances.

**Figure 10:**
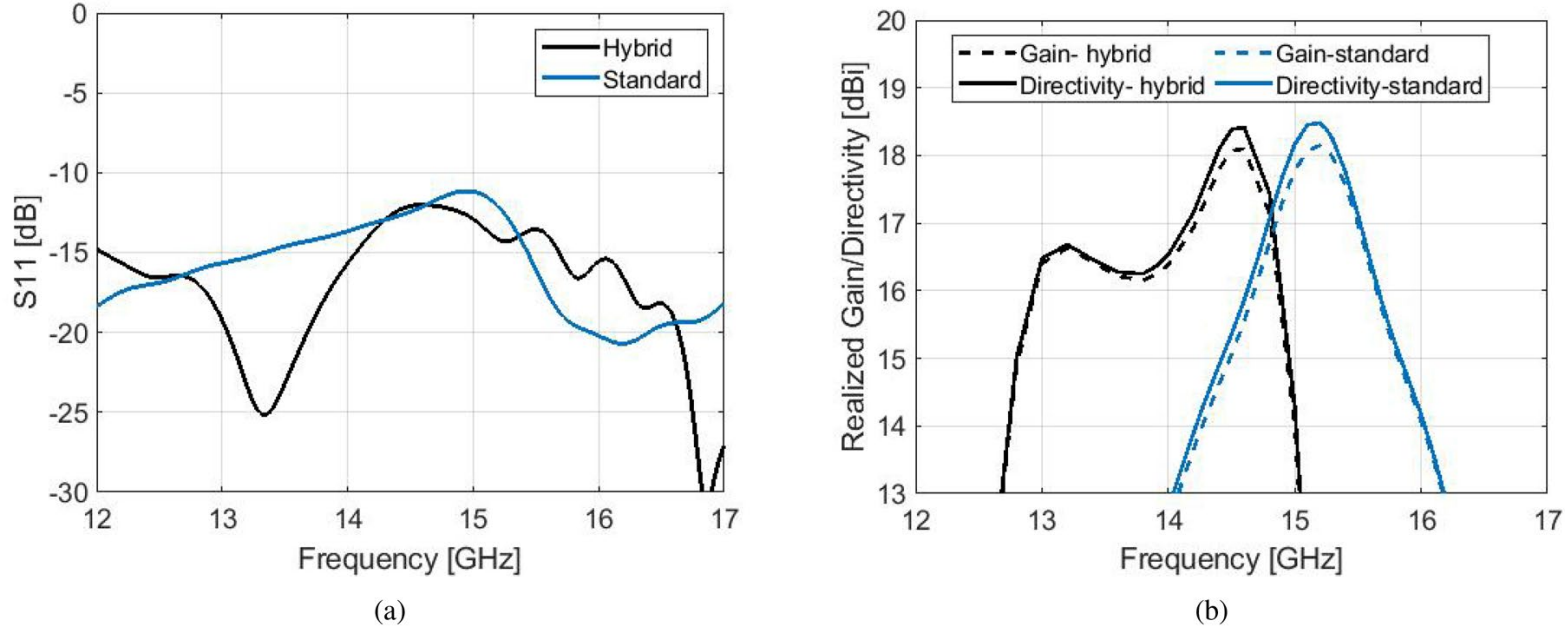
Simulation results of an optimized hybrid Bull’s Eye antenna with enhanced 3-dB gain bandwidth and comparison with a standard Bull’s eye antenna ($N=5$) with similar directivity/gain. (**a**) $S_{11}$. (**b**) Directivity and realized gain.

#### Optimum hybrid antenna with enhanced 3-dB gain bandwidth (optimum B)

The second optimum prototype presented in this work, is a $N=5$ antenna with significantly enhanced 3-dB gain bandwidth. The optimized details of this proposed hybrid antenna are $w_{r}=8$ mm, $h_{r}=1.5$ mm, $h_{b}=2.3$ mm, $w=9.5$ mm, $h=3.31$ mm. Figure 10 shows the simulated results for this antenna. The maximum gain of 18.1 dBi is achieved at 14.6 GHz, along with an extended 3-dB gain bandwidth of 15.2%. Two peaks are observed in the gain plot, each deriving from the perturbed and the double corrugation respectively.

The standard antennas of Fig. 2 cannot achieve peak gain over 17.7 dBi. Therefore, for a proper comparison with our proposed hybrid model, a standard antenna of $N=5$ was redesigned in order to achieve a peak gain of 18.1 dBi at the Ku band. with design parameters as $d=18.6$ mm, $h=3.9$ mm and $w=12$ mm. A comparison between the proposed broadband prototype and the standard antenna can be viewed in Fig. 10. The standard antenna achieves maximum gain of 18.1 dBi at 15.2 GHz, however its 3-dB gain bandwidth is only 8.6%. Our proposed model can achieve a very high gain along with a compact size and an extended 3-dB corresponding bandwidth.

**Figure 11:**
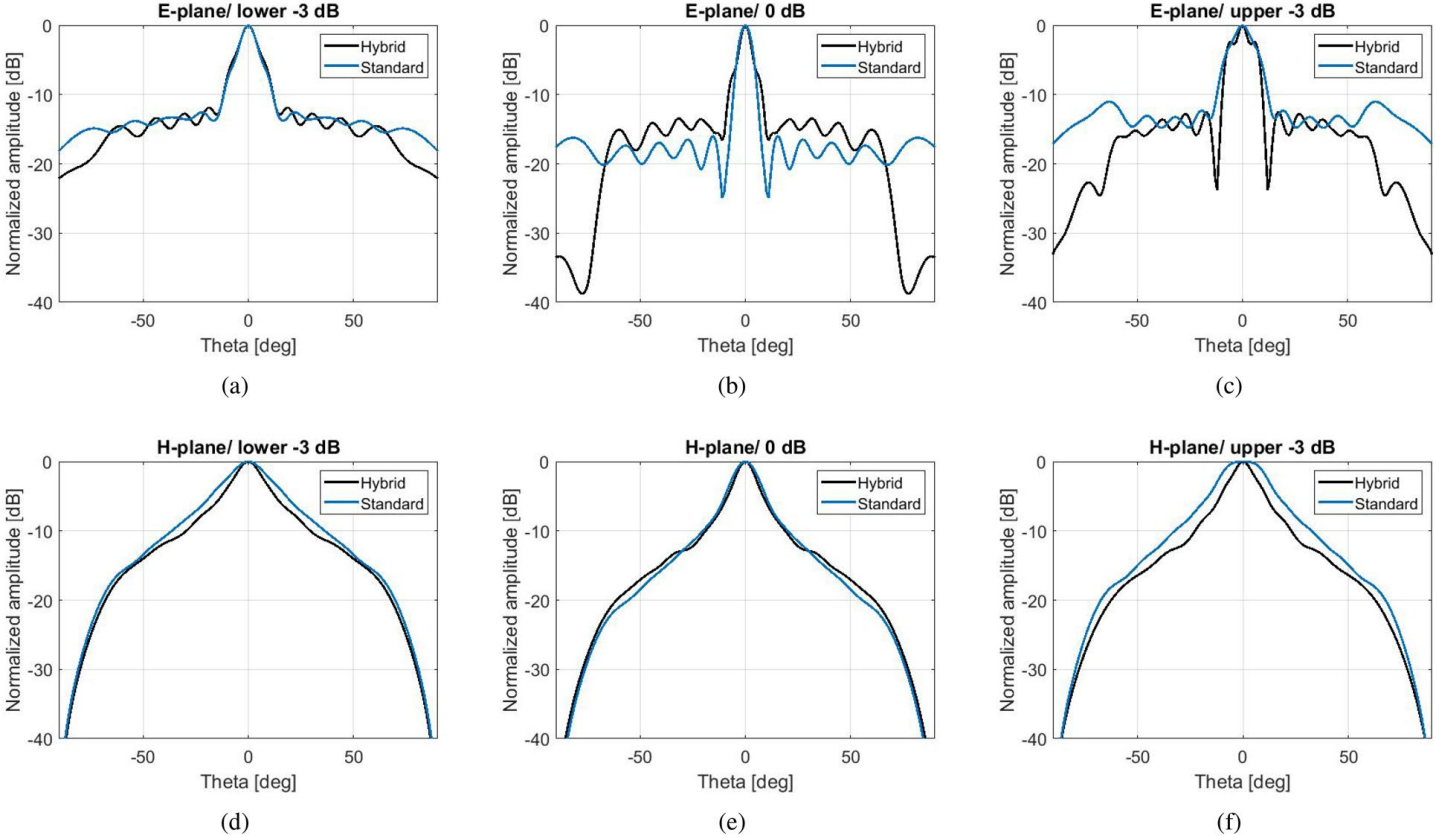
Simulated radiation patterns of the hybrid antenna with enhanced 3-dB gain bandwidth and the standard antenna of similar maximum gain.

The simulated radiation patterns of these antennas are available in Fig. 11, at the frequencies which determine the 3-dB gain spectrum. For the hybrid antenna, these frequencies are: lower − 3 dB at 12.8 GHz, 0 dB at 14.6 GHz and upper − 3 dB at 14.9 GHz. For the standard antenna, they are: lower − 3 dB at 14.5 GHz, 0 dB at 15.2 GHz and upper − 3 dB at 15.8 GHz. At the frequency point of maximum gain, the side lobe level of the *E*-plane is at − 13.5 dBi for the hybrid antenna and − 16 dBi for the standard antenna, maintaining a highly directive lobe at broadside. Similarly to the comparison in the previous section, at bigger angles (over $\pm 60^{\circ }$) the hybrid antenna performs better (side lobe level way below − 20 dBi). At the limits of the 3-dB spectrum the hybrid antenna performs better than the standard antenna for all $\theta$.

## Discussion

In this work, the typically narrowband gain performance of the standard Bull’s Eye antennas is extended with the use of novel types of corrugations. The perturbed and the double corrugation concept offer elements of bandwidth enhancement while maintaining a high gain. Their combined effect into a hybrid shape achieves a significant expansion of 1-dB and 3-dB fractional gain bandwidth compared to corrugated antennas of similar size and gain. After an extensive simulation study of the perturbed and double corrugations, both separately and into a hybrid form, a design methodology can be summarized as following:

- An increase of height $h_{b}$ of the perturbed corrugation results in a drastic increase of the maximum realized gain for gap widths $w \ge \lambda _{0}/2$. The dimension $l_{b}$ does not affect the performance of the antenna. For an effective trade-off between maximum gain and gain bandwidth, it is suggested that $h_{b}$ is set between *h*/2 and 2*h*/3.
- As expected from Eq. 1, with an increase in $h_{r}$, the central frequency of operation is shifted towards lower frequencies. The effect of the set of $w_{r}$, $h_{r}$ is observed only for $w \le \lambda _{0}/2$. For an enhancement in peak gain and gain bandwidth, the suggested optimization boundaries should be set at $h_{r}<h/2$ and $w_{r} \sim w$.

**Figure 12:**
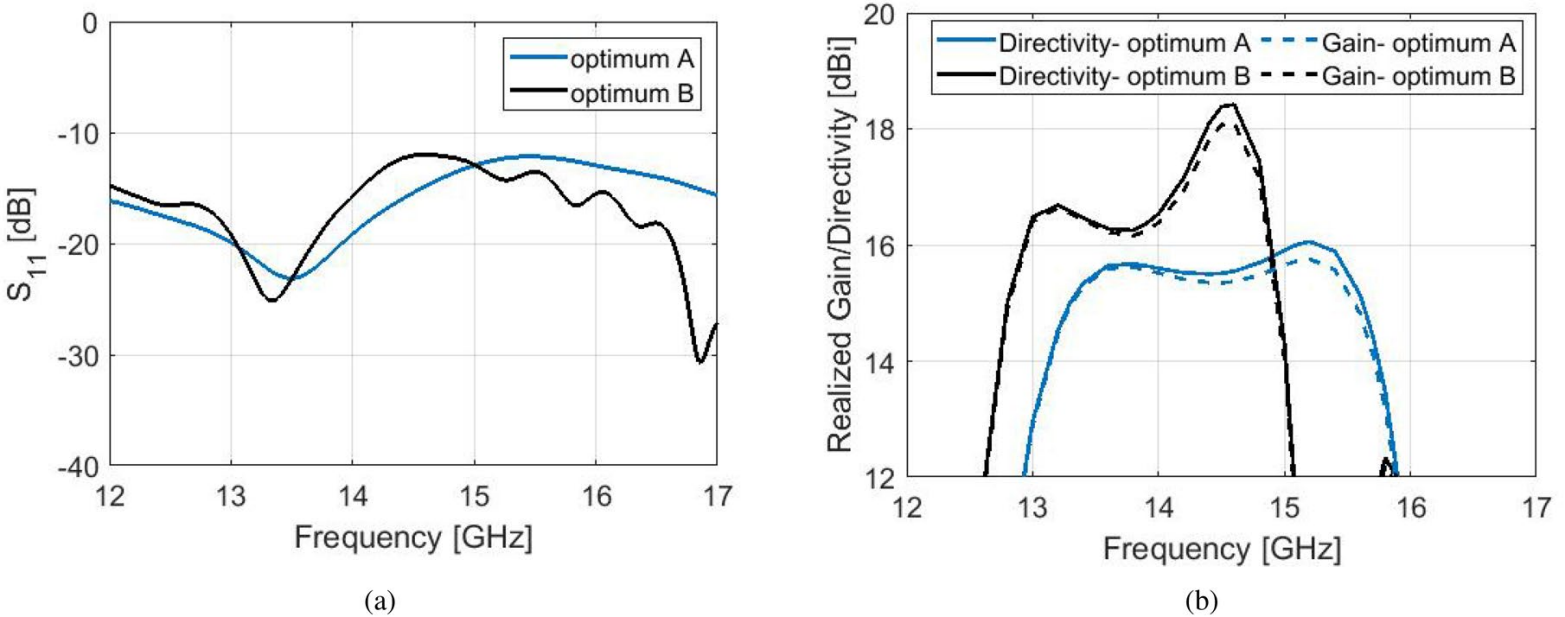
Comparison of simulated results between optimum A and B. (**a**) $S_{11}$. (**b**) Directivity and realized gain.

The optimization of process of the hybrid corrugated unit cell generated numerous designs with improved the gain bandwidth performance. The antenna could be alternatively optimized with the use of state-of-art evolutionary algorithms, or algorithms that solve the so-called inverse design problem.The presented designs were selected either due to their extraordinary flat gain performance over an enhanced bandwidth, or their increased 3-dB gain bandwidth at such high gain values (over 18 dBi). Simulated results demonstrate an increase of 7.3% in 1-dB bandwidth and 2.2% in 3-dB bandwidth for the flat-gain prototype, compared to a standard Bull’s Eye antenna of the same size and gain. Additionally, the proposed hybrid model of maximum gain at 18.1 dBi achieves a 6.6% increase in 3-dB gain bandwidth, compared to a standard antenna of same size and gain at similar frequencies. A comparison between our two proposed designs can be viewed in Fig. 12 in simulated $S_{11}$, realized gain and directivity.

The proposed hybrid shaped unit cell achieves a bandwidth expansion without adding to the overall antenna size, either by increasing the size of the antenna in rings, or, by superimposing dielectric layers^7^ or corrugated layers^8^. The advantages of corrugated metallic antennas, that include high gain, easy design process and simple feeding network are maintained in the hybrid corrugated implementation. Still, as a future expansion of this work, the proposed hybrid unit cell could be used in combination with the aforementioned techniques, or other bandwidth enhancement techniques^15^ for a further optimized performance.

A hybrid corrugated prototype can be easily fabricated with CNC technique at microwave and lower millimeter wave frequencies, as the proposed design methodology is directly scalable to higher frequencies. A similar response can be expected in maximum gain, directivity, $S_{11}$ and fractional 3-dB gain/directivity bandwidth, after a minor optimization process. While a CNC milling fabrication of such a prototype at low THz would remain challenging with the current limitations of the fabrication tools, in the future an improvement of ultra-high precision tools and cutters could enable a fabrication with this technique. Other fabrication methods, such as 3-D printing^16^ or metal 3-D printing^17^ could be employed at higher mm-wave frequencies.

## Methods

All the antennas presented in this work were designed and simulated with the use of a full-wave simulation software (CST Microwave Studio). Open boundaries were used for the simulation of the antennas and periodic boundaries for simulations at the unit cell level.

The selected feeding waveguide (WR-62) operates between 12.4 GHz and 18 GHz. For the fabrication of the antenna prototype, the sharp rectangular edges of the open-ended waveguide aperture were rounded in order to facilitate the fabrication process. From simulations, such an alteration was not found to affect the performance of the antenna. The fabrication of the prototype was made with CNC milling technique at the University of Birmingham. Holes were drilled at the rear of a selected aluminium antenna body for the fitting of the screws of the WR-62 waveguide flange.

The measurement of the prototype took place in an anechoic chamber at the University of Birmingham, with the use of a ZVA65-A VNA. The realized gain of this antenna was measured with the use of two identical broadband horn antennas, operating between 2 and 18 GHz, with approximately 12 dBi realized gain over this bandwidth. The realized gain of the prototype was calculated after the measurement of the $S_{21}$ parameters between the prototype and the horn antennas, with the use of the substitution method^18^. The prototype was placed at a distance of approximately 4.5 m from the transmitter horn antenna (within the farfield region of the Bull’s Eye antenna), according to the formula $d \ge 2D^{2}/ \lambda _{0}$ where *D* is the diameter of the Bull’s Eye antenna. For the measurement of the radiation patterns, the prototype was mounted onto a rotating base, while one horn antenna was stable at the selected distance of 4.5 m. Both *E* and *H* planes were measured by rotating the two antennas so that their respective planes were aligned. The resolution of these patterns is $1^{\circ }$.

Although the $S_{11}$ and radiation pattern measurements were successful, there is about 1.5 dB loss in the gain measurement between 14.3 and 15 GHz. This mismatch is attributed to errors during the fabrication process. However, the overall measured gain performance remains within the simulated 3-dB gain bandwidth.
